# Toughness Evolution of Flax-Fiber-Reinforced Composites under Repeated Salt Fog–Dry Aging Cycles

**DOI:** 10.3390/polym16131926

**Published:** 2024-07-06

**Authors:** Luigi Calabrese, Carmelo Sanfilippo, Antonino Valenza, Edoardo Proverbio, Vincenzo Fiore

**Affiliations:** 1 Department of Engineering, University of Messina, Contrada Di Dio (Sant’Agata), 98166 Messina, Italy; lcalabrese@unime.it (L.C.); eproverbio@unime.it (E.P.); 2 Department of Engineering, University of Palermo, Viale delle Scienze, Edificio 6, 90128 Palermo, Italy; carmelo.sanfilippo01@unipa.it (C.S.); antonino.valenza@unipa.it (A.V.)

**Keywords:** flax fiber, environmental degradation, repeated cycles, salt fog, drying, moisture desorption

## Abstract

This research examined the response of flax-fiber-reinforced composites (FFRCs) to simulated outdoor conditions involving repeated exposure to salt fog and drying. The study investigated the effect of cycles on the toughness of the FFRCs. To achieve this, the composites were exposed to humidity (salt fog) for 10 days, followed by 18 days of drying in cycles. A total of up to 3 cycles, each lasting 4 weeks, were conducted over a 12-week period. Throughout this process, changes in the material’s weight, water absorption, and mechanical properties were monitored by water uptake and three-point bending tests. The findings revealed the significant impact of these humid–dry cycles on the mechanical response of the FFRCs. When exposed to humid environments without drying, the composite’s toughness increased significantly, due to a weakening effect more pronounced for stiffness, with strength reductions of about 20%. However, subsequent drying partially restored the material’s performance. After 18 days of drying, the composite regained most of its initial performance.

## 1. Introduction

The field of natural fiber-reinforced composites (NFRCs) has acquired an increase in interest in recent years, thanks to their potential to offer a compelling combination of desirable mechanical properties and environmental sustainability [1,2,3]. NFRCs address the growing need for robust, long-lasting materials that align with eco-friendly practices [4]. Unlike conventional composites, which rely on synthetic fibers, NFRCs utilize renewable plant fibers such as flax, hemp, jute, and kenaf, contributing to a more sustainable production model [5].

Natural fiber reinforced composites display acceptable tensile/flexural strength and stiffness, extending their use for semi-structural applications [6,7,8]. Furthermore, given their low density, natural fibers contribute to the overall lighter weight of the composite [8,9,10]. Additionally, because they form a natural and renewable resource, natural fiber NFRCs have a lower environmental footprint than their synthetic counterparts.

While natural fiber reinforced composites offer some structural and sustainability advantages, their widespread use is hindered by inherent drawbacks. These materials often exhibit poorer interfacial adhesion between the fibers and matrix when compared with synthetic composites [11,12]. This weak link can lead to reduced mechanical strength and increased moisture absorption. The hydrophilic behavior of lignocellulosic fibers makes them susceptible to water uptake, causing dimensional instability and promoting mechanical degradation [3,13]. Therefore, a key challenge associated with NFRCs is their susceptibility to moisture, which limits their use indoors and restricts them mainly to the outdoors, where fluctuating humidity is common, in turn hindering their broader adoption.

Though there is a wide literature examining the behavior of natural fibers and their composites in humid or alternate environments, research on the recovery of these materials following humid–drying stages is limited. Further exploration into this aspect could provide valuable insights for the development of more robust and resilient composite materials in various environmental conditions.

Outdoor environments often expose materials to fluctuating humidity levels, raising concerns about the long-term performance of natural fiber composites. Coastal regions, for example, subject materials to cycles of salt spray and drying due to sea spray and varying weather patterns. Similarly, outdoor applications experience seasonal changes in humidity that can lead to repeated moisture absorption and desorption. Understanding how these cycles affect NFRPs’ performances is crucial for ensuring their durability and reliability in service life.

Lu and Van Vuure [14] investigated the impact of using dry vs. non-dry flax fibers (conditioned at varying humidities) on the moisture resistance of flax/polyester and flax/epoxy composites. Composites with non-dry fibers showed better performance throughout long-term moisture cycling compared with dried fibers. Unexpectedly, all composites exhibited improved flexural properties after cycling, likely due to combined stiffening of resins and flax fibers.

Furthermore, Kim and Seo [15] examined the impact of water absorption cycles on tensile and fracture toughness of woven sisal composites in epoxy and vinyl-ester matrices. Specimens underwent 5 cycles of immersion and drying (9 days of immersion in water followed by 1 day of drying at 50 °C), revealing decreased mechanical properties with increasing water exposure.

Van Schoors et al. [16] analyzed the effect of year-long cyclic moisture on the transverse properties of unidirectional flax–epoxy composites. Their results highlight the role of the matrix and interface in degradation. Moisture-induced weakening, including tensile strength loss and modulus drop, is likely to be due to debonding and matrix plasticization.

A further study, by the same research group [17], assessed how year-long humidity fluctuations, simulated with alternating high (90%) and low (40%) humidity cycles at elevated temperature (55 °C), impacted the strength and stiffness of a flax/epoxy composite. While their analysis identified some fiber and interface weakening, the effect was minor (strength and stiffness reduction below 15%). This suggests that well-manufactured flax composites can endure repeated moisture exposure, opening doors for broader applications.

Mak et al. [18] highlighted the way in which the tensile strength of flax-fiber-reinforced polymers (FFRPs) increased in wet conditions but degraded after multiple wet–dry cycles. Despite the glass-fiber-reinforced polymers (GFRP) remaining unaffected, FFRPs showed faster degradation.

Our previous studies [19,20] investigated how a flax fiber composite regains performance after repeated exposure to a marine environment (salt fog for 15 or 30 days followed by dry storage). We found that the material’s properties suffered both reversible and irreversible modifications during the wet stage, but significantly recovered during the drying stage. This suggests that strength loss is temporary, while stiffness loss is more permanent. Both factors influence a composite’s toughness during wet–dry cycles.

Hence, the importance of understanding how flax fiber composites perform and degrade under humid and dry conditions (hydrothermal aging) is growing, as evidenced by the increasing research in this area. However, a key gap exists in terms of the reversibility of these composites’ performance after repeated cycles of salt spray and drying.

Addressing this knowledge gap and relating such a finding to the mechanical and toughness performance of the NFRC would significantly enhance the value of research in this field.

Given this, the aim of this work is to study the impact of alternate salt fog/dry aging cycles on the mechanical performances of flax-fiber-reinforced epoxy composites. Salt fog environments simulate marine or coastal conditions, where composites experience moisture absorption and potential degradation. The subsequent dry phase allows for partial performance recovery.

According to our previous paper [21], the aging time for the composite laminates was 12 weeks, involving the following humid–dry cycles that lasted 4 weeks each: 10 days humid, 18 days dry. In the humid phase, salt spray fog (5 wt.% NaCl, 35 °C, 95% RH) was used based on ASTM B 117 standards [22], using an Ascott CC1000iP corrosion chamber. Dry phase conditions were maintained at 22 °C, 50% RH (atmosphere class 2, according to ISO 291 standard [23]). Three-point bending tests were conducted at increasing dry time (0, 5, 10, and 18 drying days) according to ASTM D790 [24] using a Universal Testing Machine (U.T.M.). Furthermore, weight monitoring was performed to assess water absorption, density and void content at intervals during both phases.

By exploring the influence of varying salt fog exposure durations and dry recovery times, this research aims to elucidate the mechanisms affecting performance degradation. The findings will provide valuable insights for optimizing the design and application of flax fiber composites in environments with fluctuating humidity. This knowledge can pave the way for the wider adoption of sustainable natural fiber composites in outdoor scenarios.

## 2. Experimental

### 2.1. Materials and Methods

Vacuum-assisted resin infusion (VARI) was employed to manufacture square flax-fiber-reinforced polymer (FFRP) laminates with dimensions of 30 cm by 30 cm and with a nominal thickness of 0.335 cm. A vacuum pump model VE 235 D from Eurovacuum (Reeuwijk, The Netherlands) was used to achieve a pressure of 0.1 atmospheres (absolute) within the vacuum bag. Each panel underwent the following two-step curing process: (i) initial curing at room temperature (25 ± 1 °C) for 24 h; (ii) post-curing at a temperature of 50 °C for 15 h. The panels utilized a DEGBA epoxy resin (SX8 EVO from Mates Italiana, Segrate, Italy) mixed with its corresponding amine-based hardener in a weight ratio of 100 to 30 parts (resin to hardener). The hardener is a modified cycloaliphatic polyamine with specific gravity, viscosity, and gel time at 25 °C of 0.96 g/cm^3^, 200 mPas, and 6 h, respectively.

This blend served as the binding agent (matrix) for the composite material. Five layers of flax twill woven fabric, supplied by Lineo (Valliquerville, France) and weighing 318 g/m^2^, were used as continuous natural fibers with which to reinforce the panels.

### 2.2. Salt Fog/Dry Aging Phases

This study investigates the effects of repeated exposure to humid and dry environments, similar to those experienced in outdoor marine applications, on the ability of flax-fiber-reinforced polymers (FFRPs) to recover their mechanical properties and related toughness after each humid cycle. Composite panels underwent a series of tests involving exposure to harsh conditions and mechanical evaluation.

The testing involved the following:

Humid cycles: Panels were sprayed with a salt solution (5% salt) for a maximum of 10 days at 35 °C in a climatic chamber (model CC1000iP by Ascott analytical, Tamworth, UK), following the ASTM B 117 standard.

Dry storage: After the salt spray, five samples were kept in a controlled environment (50 ± 10% relative humidity, 22 ± 2 °C, according to ISO 291 standard) for up to 18 days before testing.

Multiple cycles: Up to 3 complete cycles of humid and dry conditions were applied. Each full cycle lasted a maximum of 28 days, combining the humid and dry phases.

The definition of the humid–dry protocol was carefully established by referencing previous experimental tests [20]. This aims to optimize the potential to reversibly mitigate the performance degradation that may occur during the aging process.

For lack of clarity, a code to name the specimens was applied. In particular, the samples were labeled as “nWaDb”, where n represents the cycle number, a represents the number of days of the salt fog exposure and b represents the number of drying days.

For instance, “3W10D3” refers to specimens which are currently undergoing their third aging cycle, have completed the salt fog exposure (i.e., 10 days) and were then stored for 3 days in a dry environment. Unaged samples (control group) were coded “W0D0”.

### 2.3. Water Changes

To investigate the link between water absorption and the duration of salt fog exposure, three square-shaped samples measuring 100 × 100 mm^2^ were periodically removed from a climatic chamber over a ten-day period. Based on the ASTM D570 standard [25], the samples were meticulously wiped with a dry cloth to eliminate any surface moisture before being weighed using a high-precision analytical balance (model AX 224 by Sartorius, Goettingen, Germany—precision 0.1 mg).

The weight change (*WC*) of the composites was calculated using Equation (1) [26]:

(1) $$WC\;\left[\%\right]=100\cdot \frac{M_{ti}-M_{U}}{M_{U}}$$

The monitoring process involved utilizing this formula to track the changes in weight for both the gain and loss experienced in humid and dry phases. *W*_0_ is the initial weight of the untreated dry sample, while *W_ti_* identifies the weight of the sample after exposure to aging time ti, which is composed of the total duration spent in the salt fog chamber and subsequent drying stages. In this way, one could compare the weight variations under different environmental conditions, and thus provide valuable insights into the material’s behavior and performance characteristics throughout the aging process.

The density of the aged composites was also determined based on water uptake results. To evaluate the density (represented by ρ_ce_) for all samples, three replicas in weight and volume measurements were performed. The measurements evidenced a low standard deviation (below 0.015 g/cm^3^).

### 2.4. Quasi-Static Three-Point Bending Tests

Five prismatic specimens measuring 13 mm by 64 mm were mechanically tested under quasi-static three-point bending conditions. The testing followed ASTM D790 standards and was conducted using a U.T.M. model Z005 (Zwick-Roell, Ulm, Germany) with a 5 kN load cell. The span distance and crosshead rate were held constant at 54 mm and 1.4 mm/min, respectively.

**Figure 1:**
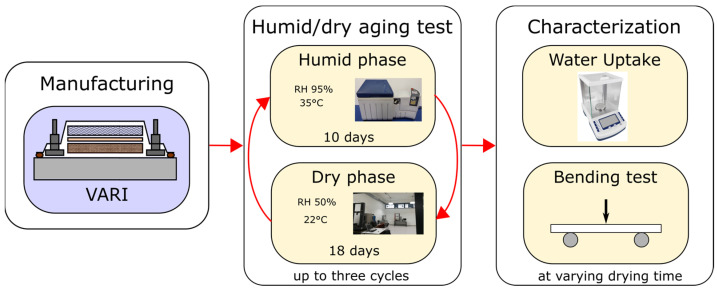
Graphical scheme of the experimental campaign.

Figure 1 graphically shows the experimental approach applied in the composite laminate. It details the composite preparation technique, the hydrothermal aging cycles used, and the key characterization methods employed, which include water uptake measurement and three-point bending tests.

## 3. Results and Discussion

### 3.1. Water Uptake Measurements

**Figure 2:**
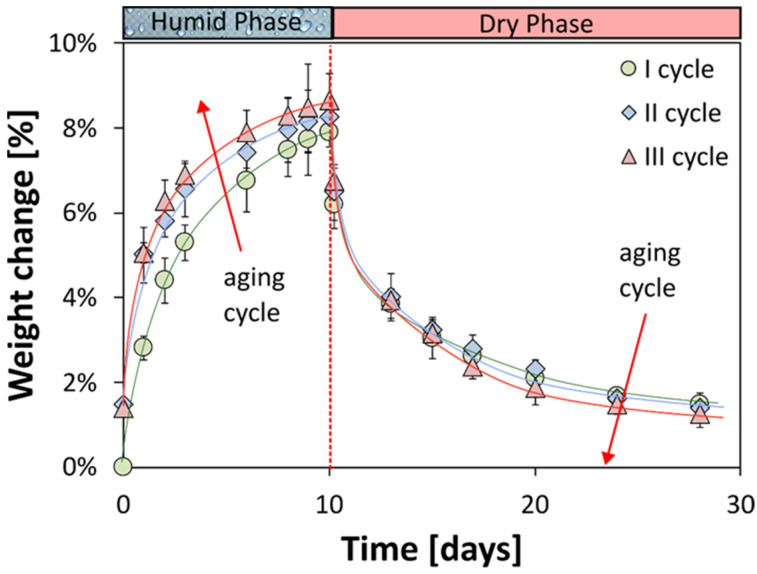
Weight change vs. time during humid and dry phases for the flax composite laminate at varying aging cycles (cycle I: circle marker; cycle II: diamond marker; cycle III: triangle marker).

Figure 2 illustrates the weight change, determined based on Equation (1), of the flax composite laminate over time (days) during the adsorption and desorption processes in both the humid and the dry cycles for the three aging cycles.

Salt fog exposure, occurring in the first 10 days of the aging cycle, causes materials to quickly absorb moisture, with rapid weight gain. It was observed that the water uptake already exceeded 4% after the first cycle in the first two days. This rate decreases as exposure continues, with a noticeable shift in the weight gain trend around 5–7 days. The material does not reach a healthy condition and this can be seen from the curve, which does not show a horizontal asymptote at the end of the 10 days of the humid phase. However, the trend of the curve does show a progressive convergence towards an asymptotic value, indicating the tendency of the material towards an equilibrium of the water absorbed during this phase.

After 10 days (240 h) of exposure in the first cycle, the flax laminate shows a maximum weight gain of 7.9%. This increase is due to several factors that make flax-fiber-reinforced composites more likely to absorb water. The materials used in the composite naturally attract water, which allows it to be absorbed on the surface. This starts the process of water uptake. Water molecules then interact with reactive groups in the epoxy resin, such as hydroxyl and amine groups, which are attracted to water and have not yet reacted. These interactions create channels that allow even more water to diffuse into the material [27,28]. The hydrophobic nature of flax fibers also plays a key role in the water uptake process [21,29]. The water-induced degradation of flax composites involves the following three stages [30,31]: (i) initial water uptake, where water molecules initially diffuse through the tiny channels and spaces on the surface of the material (matrix); (ii) capillary-driven water transport, where, as water enters, capillary forces pull it further into the material, increasing the rate of diffusion; and (iii) material breakdown, where, over time, factors like fiber swelling and matrix softening (caused by aging) lead to the separation of the fibers from the matrix and the formation of cracks within the matrix [32]. Additionally, water weakens the flax fibers by disrupting the bonds within them, creating easier pathways for even more water to enter [33].

Therefore, several competing processes, happening at the same, contribute to the weight gain in the natural composite laminate, involving a bimodal curve trend deviating from the expected behavior (i.e., Fickian) that is due to the permanent damage and continuous water uptake incurred by increasing humidity time exposure [34].

Similarly, during the dry phase, FFRP exhibits a progressive decrease in weight. Mirroring the absorption phase, the weight rapidly decreases during the early stages of drying and then gradually stabilizes over longer drying periods. Notably, during the first cycle, a weight reduction above 4% was observed after 3 days of exposure to 22 °C and 50% relative humidity, indicating a rapid initial desorption. After approximately 7 days (10 humid days + 7 dry days), the curve deviates from linearity to approach an asymptotic value. By the 18th day of drying in the first cycle, the composite’s residual weight had changed by 1.47%.

The weight change of the FFRP material throughout humid–dry cycles showcases the interesting and consistent trends over multiple cycles. It is noteworthy that the weight tends to increase during the humid phase and decrease during the dry phase, illustrating a recurring pattern. Though there may be slight variations in the intensity of weight gain and loss across cycles, there is a distinct overall trend towards greater weight gain. For instance, in the second and third cycles, a visible increase in the maximum weight gain percentages can be observed, reaching 8.25% (at the end of the humid phase in the second cycle) and 8.65% (at the end of the humid phase in the third cycle). Moreover, as the cycles progress, there is a slight decrease in the weight remaining at the end of the dry cycle. In fact, in the second cycle, this value reaches 1.38%, while in the third cycle it stands at 1.25% weight gain. These data reveal a cumulative effect where the material retains and releases more moisture over successive cycles, emphasizing a gradual change in water absorption and desorption behaviors within the FFRP material. It implies that, with an increase in cycle count, the difference between the minimum and maximum weight during humid and dry phases, denoted as the gap between the maximum and minimum values of the weight gain, also expands, indicating a growing tendency for water absorption and release in the material that influences its overall weight fluctuation dynamics. This behavior can be ascribed to the triggering of degradation phenomena on the material induced by the different adsorption and desorption cycles (constituents dissolution, swelling, debonding, cracks and so on) [35]. Following the diffusion of water, presence paths are formed in the constituents of the composite and their interface. The latter favors the further diffusion of water during the increase in adsorption cycles. At the same time, the same paths generated during the aging cycles allow easier evaporation of the adsorbed water, with a consequent reduction in the residual weight change at the end of cycle III. The observed ability of the material to release most of the absorbed water during a short drying period suggests that this water uptake is primarily due to a reversible physical adsorption process [36]. However, the presence of a small persistent weight change even after the third cycles indicates that irreversible alterations, potentially related to permanent degradation phenomena, may have also occurred within the material during the humid phases [20].

To gain a deeper understanding of how water absorption influences material aging, changes in both the bulk density and void content of the composite materials were monitored over time during the humid and dry phases of the experiment (Table 1). This comprehensive investigation aims to better reveal potential correlations between the amount of water uptake and changes in the material’s internal structure and density. The void content was determined based on the apparent density and the bulk density of the composites. Standard deviation was also reported for all measurements.

Composites achieve higher density at increasing exposure time in the salt fog chamber. This behavior is related mainly to flax fibers, which strongly attract water due to their hydrophilic behavior and thus readily favor the penetration of water to composite layers [37]. Additionally, the weak bond between the flax fibers and the hydrophobic epoxy matrix further facilitates water absorption at the interface. This increase in density is linked to a decrease in voids, which can be verified through analysis. During the humid phase, the water penetrates the composite structure, filling previously existing empty voids [38]. At the same time, material swelling also acts to reduce the volume of voids and cavities in the composite bulk. This initial increase in density is followed by a decrease during the drying phase. This decrease likely occurs because the previously absorbed water evaporates, leaving behind empty spaces or cracks within the composite generated e.g., by differential matrix–fiber shrinkage or fiber–matrix debonding.

In summary, after three humid–dry cycles, the FFRP reports respective density values of 1.128 g/cm^3^, 1.125 g/cm^3^, and 1.109 g/cm^3^. Correspondingly, the residual void contents are 5.97%, 6.30%, and 7.61%, respectively. Thus, interestingly, at an increasing number of aging cycles, the difference between the density and void content in the humid and dry states became more significant. This suggests that repeated exposure to humid and dry conditions accelerates the creation of defects and voids within the composite, potentially triggering permanent degradation processes. No significant changes in standard deviation can be identified in density and void content at varying humid–dry cycles, indicating an almost compatible distribution of data.

### 3.2. Three-Point Bending Tests

**Figure 3:**
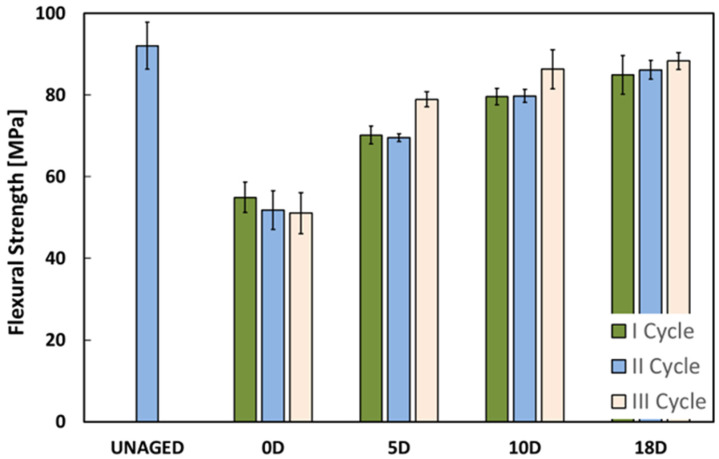
Histogram of the evolution of the flexural stress at increasing drying time for the 1st, 2nd and 3rd cycles.

**Figure 4:**
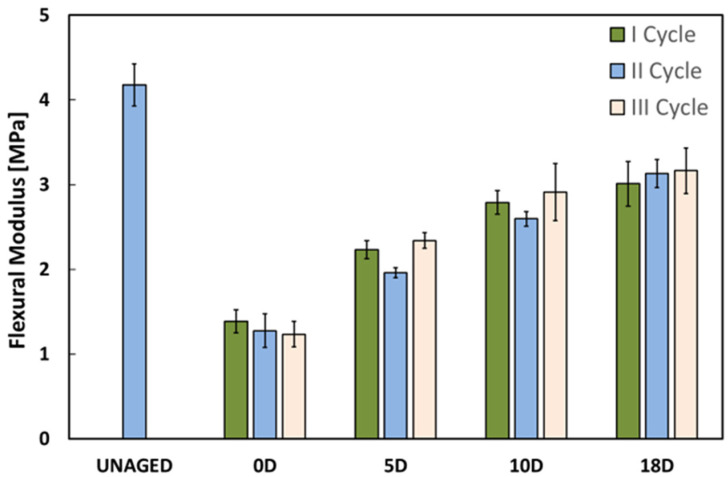
Histogram of the evolution of the flexural modulus at increasing drying time for the 1st, 2nd and 3rd cycles.

Figure 3 and Figure 4 show the trend of the flexural strength and modulus and the variation of the drying time for all the aging cycles, respectively. By analyzing the histogram concerning the strength average values (Figure 3), it can be seen that the mechanical resistance of FFRPs is significantly influenced by the imposed aging conditions.

At the end of the humid phase, the specimens that have not yet undergone exposure to a drying cycle (0D samples in Figure 3) show a clear reduction in mechanical resistance compared with the unaged specimens. The exposure to the humid environment (although for a limited period of 10 days) was enough to trigger weakening phenomena and performance decay, leading to a reduction in mechanical resistance from 92.1 MPa for the unaged specimen (UNAGED) to 51.1 MPa at the end of the third wet cycle (III cycle, 0D class in Figure 3). This indicates that a percentage reduction of 44.5% has taken place.

Likewise, the mechanical strength is progressively lower as the number of cycles increases. In fact, the specimens show gradually lower resistance values during the first, second and third cycle, confirming that exposure for prolonged periods to alternating wet–dry cycles can have detrimental and harmful effects on the performance of FFRPs, which show an increasingly greater degradation sensitivity to humid environment.

However, the degradation phenomena occurred during the humid phases (causing the aforementioned strength reduction) do not show a permanent effect on the composite. In fact, NFRP material highlights a gradual recovery of performance during the drying phases of each aging cycle. Regarding the first cycle, the composite is already able to recover approximately 28% of the lost strength after 5 drying days (5D samples in Figure 3), going from 54.9 MPa to 70.2 MPa. The phenomenon recovery continues to occur even at longer drying times. By the end of 18 days of drying (18D sample) the material has almost completely recovered its initial resistance before aging, regardless of the number of aging cycles. Furthermore, it is possible to notice that, although the results at the end of the drying phase are statistically quite similar, analyzing the average values reveals a trend of increasing maximum residual resistance with a higher number of cycles.

Similar considerations can be drawn by analyzing the trend of the flexural modulus as the drying time varies (Figure 4). In this case the impact of the aging conditions on the stiffness of FFRPs is more relevant than the bending resistance. Exposure to a humid environment, even for short durations, can trigger evident softening and plasticization of both the natural fibers [39] and the epoxy matrix [40], thereby reducing the composite’s stiffness [41].

This effect can be partially ascribed to the plasticization phenomena of the epoxy matrix. Furthermore, the high hydrophilicity of flax fibers, which facilitates the absorption and diffusion of water through the composite. This promotes the dissolution of lignin and hemicellulose from the natural fiber itself, which leads to a decline in the fiber–matrix stress transfer capability and a consequent worsening of their interface between fiber and matrix [42]. In addition, the hydrophilic nature of natural fibers causes flax fibers to swell significantly more than the surrounding epoxy matrix. Hence, this differential swelling generates a radial stress at the fiber–matrix interface, potentially leading to interfacial micro-cracks in the matrix and local fiber–matrix debonding [31].

The drying step of each cycle partially mitigates this effect by allowing a significant recovery of the modulus, going, for the first cycle, from 1.39 GPa (I cycle, 0D samples in Figure 4) to about 3 GPa (I cycle, 18D), leading to an increase of 116%.

These findings indicate a considerable impact of the selected aging conditions on the overall performance and durability of the composite material, highlighting the necessity to further assess comprehensive approaches and optimization strategies by which to enhance the material’s mechanical properties under alternate environmental aging conditions.

**Figure 5:**
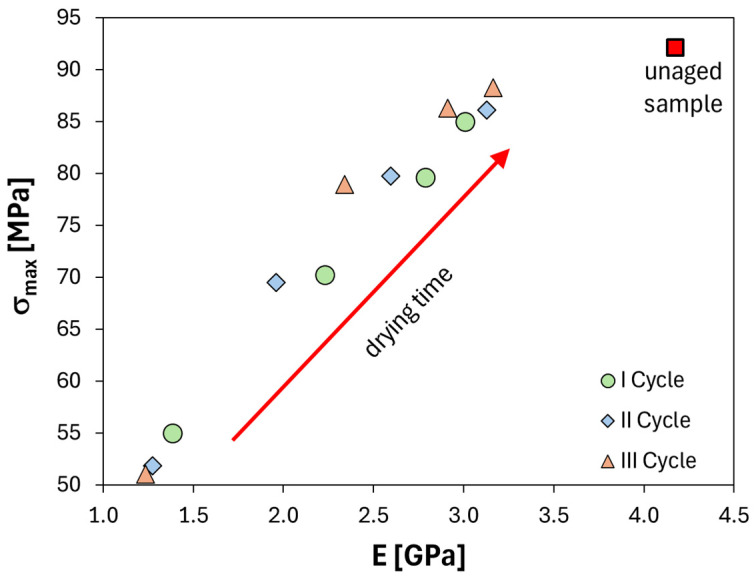
Maximum stress versus modulus plot at increasing drying time for the 1st, 2nd and 3rd cycles (cycle I: circle marker; cycle II: diamond marker; cycle III: triangle marker).

In this context, to better explain the effect of alternating humid–dry cycles on the flexural properties of FFRP material, the maximum stress versus modulus plot at increasing drying time for the first, second and third cycles is reported in Figure 5. The red square marker identifies the unaged specimens (i.e., characterized by strength and modulus average values equaling 92.1 MPa and 4.2 GPa, respectively).

By observing this graph, it is possible to notice a linear relationship between a material’s stiffness (i.e., modulus E) and its maximum bending stress (σ_max_). As drying time progresses, both mechanical parameters tend to increase monotonically. However, upon reaching the end of the drying phases, the improvement in stiffness is significantly lower than the improvement in strength, as confirmed by the larger residual modulus gap compared to the residual strength gap. This can be quantified by their respective final values: 3.2 GPa for modulus and 88.3 MPa for strength, with the former slightly lower than the unaged one (4.18 GPa). This graphically confirms a more pronounced effect of the drying process on regaining the material’s maximum resistance (i.e., flexural strength) compared to its stiffness (i.e., flexural modulus), thus clarifying a softening effect on the mechanical performances of the FFRP due to the applied alternate aging cycles [20].

**Figure 6:**
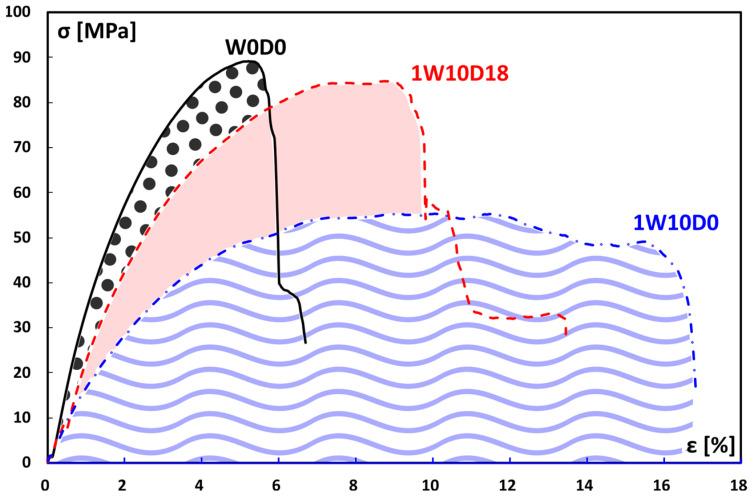
Stress–strain curves at different aging conditions in the first cycle (W0D0: unaged; 1W10D0: 0 drying days; 1W10D18: 18 drying days).

The mechanical stability of the FFRPs under alternating salt fog/dry environmental conditions can be further assessed by comparing the typical stress–strain curves of unaged (W0D0) and aged samples (1W10D0 and 1W10D18), as shown in Figure 6.

By comparing the above curves, we can immediately observe the effects of both the salt fog exposure (1W10D0) and the subsequent drying (1W10D18) on the mechanical behavior of the first-cycle aged FFRP samples. A similar finding was obtained for the second and the third aging cycle.

1W10D0 aged samples exhibited markedly decreased flexural strength and stiffness (i.e., slope of the curve) due to their exposure to the salt fog environment. They also acquired an elastoplastic behavior, indicated by the increased deflection values reached by these samples before the final failure. Furthermore, the sudden and catastrophic failure shown in unaged samples becomes progressive at higher strain values, indicating that NFRP samples acquire a ductile-like behavior after the initial humid phase [43].

Additionally, storing samples in dry conditions allows them to partially regain their flexural maximum resistance and modulus, while a noticeable reduction in the strain at break is also achieved (1W10D0 curve in Figure 6). These findings clearly show that the degradative phenomena experienced by FFRPs during the salt fog exposure (i.e., plasticization and softening) are, at least partially, recovered during the subsequent drying.

To assess the impact of moisture cycles on the laminate’s mechanical stability, we can evaluate its toughness under both humid and dry conditions. The toughness reflects the energy that materials (in this case FFRPs) can absorb before failure. As reported in [44,45], this property is proportional to the area under the flexural stress–strain curve. Consequently, tougher materials exhibit higher strength and/or larger strain at break values.

**Figure 7:**
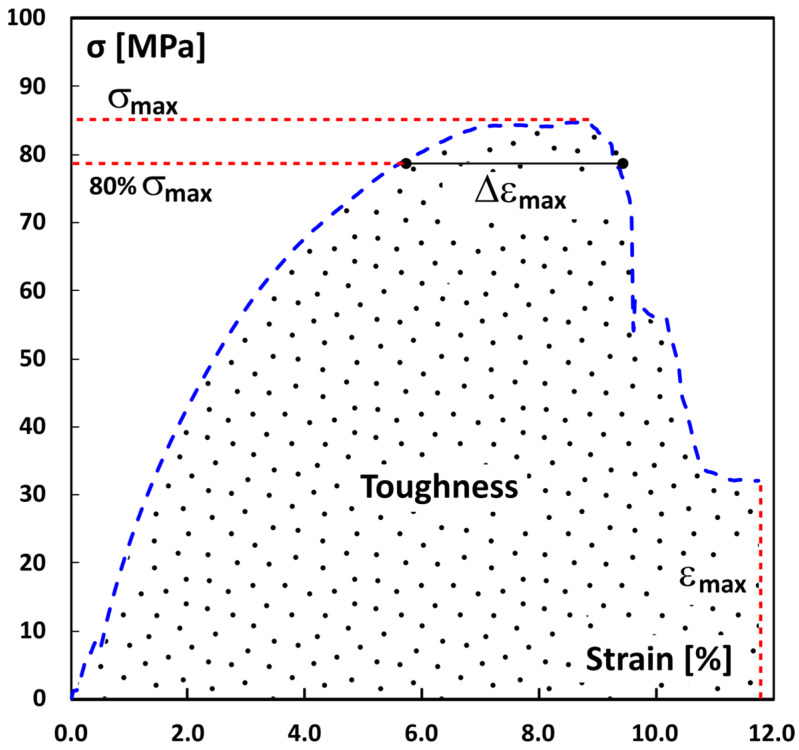
Scheme of simplified trapezoidal toughness area from stress–strain curve.

Following this concept, key parameters can be identified from the typical stress–strain curve, as shown in Figure 7.

The area under the curve can be approximated by a trapezoid, with the height and the longer base representing the maximum stress (σ_max_) and the strain at break (ε_max_), respectively. Additionally, a new parameter (Δε_max_) can be introduced. This is defined as the difference between the strain values reached at 80% of the maximum stress (representing the minor base of the trapezoid).

**Figure 8:**
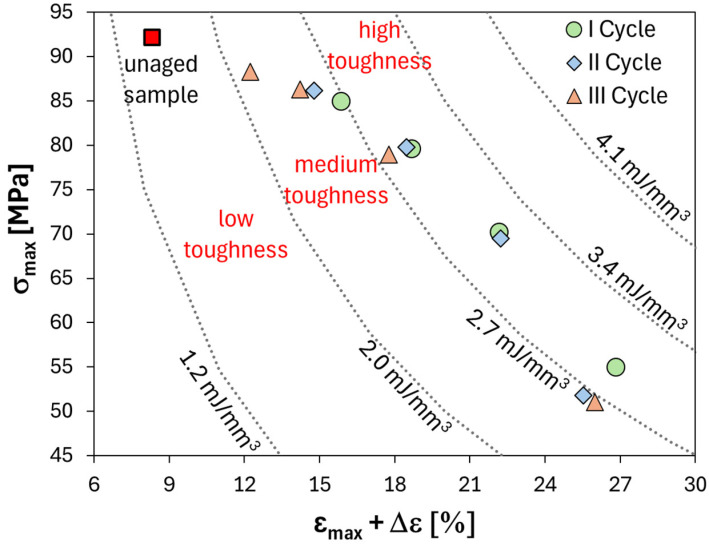
Topological map of the FFRP toughness for all aging conditions investigated.

Figure 8 shows a topological representation of the material toughness related to its strength and ductility. This graph plots “ε_max_ + Δε_max_” on the *x*-axis and “σ_max_” on the *y*-axis. By analyzing this plot, we can assess how strong and ductile the material is and, indirectly, how tough it is.

As the *x*-axis represents the sum of the trapezoid’s bases (i.e., longer plus minor) that underlies the stress–strain curve, and the *y*-axis represents the height of the trapezoid, iso-toughness curves (curves with by equal toughness value) can be determined by calculating the area of the trapezoid. Indeed, several gray dotted lines, representing equal toughness levels, can be drawn in Figure 8. These lines divide the graph into several regions characterized by different toughness. Plotting the experimental data on this graph allows one to monitor the toughness of FFRP samples under each aging condition investigated.

The graph shows that unaged FFRP (represented by the red square marker) is characterized by a mainly brittle behavior. Indeed, it has high stress but breaks at low strain, resulting in low toughness. Salt fog exposure increases toughness thanks to a relevant increase of (ε_max_ + Δε_max_), despite the reduction in peak stress due to the weakening incurred during the humid phase. No difference is seen in humid samples during all cycles (lower right corner). Salt fog for 10 days softens and weakens the material, but the overall toughness remains similar for all samples. The curves’ evolution with drying time provides valuable insights. In particular, longer drying times lead to progressive increases in both the stiffness and strength of the samples, as clearly evidenced by higher stress (*y*-axis in Figure 8) and lower deformation values (*x*-axis in Figure 8). However, the third cycle batch permanently deforms less (shifted leftward in Figure 8) compared with the first two cycles, even after drying. This means that 3W10 samples largely recover strain along *x*-axis during the dry stage compared with other batches.

Due to lower softening in the 3W10 composite, this batch demonstrates reduced toughness. Interestingly, after the 18-day dry stage, the 3W10D18 samples present similar maximum stress values to the unaged ones. However, its higher ε_max_ + Δε_σmax_ value leads to greater residual toughness of ~100% for the batch exposed to cycles of salt fog and drying compared with the unaged one.

These results show a clear link between the environmental aging and the mechanical behavior of the natural fiber composite (in terms of strength, stiffness and toughness). Notably, the toughness of the composite is highly sensitive to moisture fluctuations, weakening and losing toughness when exposed to humid–dry cycles. Future research will focus on better understanding the degradation of moisture changes and their impact on the composite’s reversible and irreversible performance. This will enhance overall understanding of this material and facilitate the design of durable NFRPs for alternating humid and dry environments.

## 4. Conclusions

This research study closely examined the weight changes and toughness evolution of flax-fiber-reinforced epoxy composites (FFRCs) under three repeated humid–dry cycles. These cycles replicated the material’s exposure to the alternating salt fog and drying conditions found in outdoor environments. The results highlighted the following:

- FFRCs undergo weight fluctuations, gaining weight during humid phases and losing weight during dry phases of each cycle. The cyclical moisture uptake weakens the material over time. The maximum weight gain increases with the number of aging cycles, reaching 8.65% in the final cycle (i.e., the third). There is a slight decrease in residual weight observed at the end of each dry phase as the number of aging cycles increases, reaching 1.25% in the third cycle. The observed ability of the material to release most of the absorbed water during drying suggests that water uptake is primarily due to physical adsorption. However, some irreversible changes likely occur within the material during humid phases.
- FFRCs exhibit an increase in density with increasing exposure time in the salt fog chamber because water molecules can fill voids within the composite structure. This increase in density is followed by a decrease during the drying phase as water evaporates. Consequently, repeated exposure to humid and dry conditions accelerates the creation of defects and voids within the composite, potentially triggering permanent degradation processes.
- The composite’s toughness is very sensitive to the humid–dry cycles. It increases due to a slight reduction on the peak stress and a large increase in the deformation at break after exposure to humid environments. Interestingly, after drying, the composite exposed to three cycles (3W10D18) showed a permanently higher toughness than the unaged composite, likely due to a larger strain at break.

Future research activities will seek to gain a more comprehensive understanding of how moisture cycles contribute to the degradation of natural composites and how this affects their reversible and irreversible performances. This examination is relevant in advancing the general comprehension of this class of materials, thereby presenting opportunities to create durable NFRCs that could withstand the challenges posed by alternating humid and dry outdoor environmental conditions.

## Figures and Tables

**Table 1:** Average density and voids content at increasing humid–dry time for the FFRPs at varying aging cycles.

	I Cycle		II Cycle		III Cycle	
Exposure Time [Days]	Density [g/cm^3^]	Void Content [%]	Density [g/cm^3^]	Void Content [%]	Density [g/cm^3^]	Void Content [%]
**Humid phase**						
**0**	1.132 ± 0.007	5.99 ± 0.5	1.128 ± 0.013	5.97 ± 0.72	1.125 ± 0.013	6.30 ± 0.49
**1**	1.16 ± 0.009	3.11 ± 0.44	1.165 ± 0.009	2.30 ± 0.52	1.16 ± 0.008	2.65 ± 0.62
**2**	1.174 ± 0.011	1.62 ± 0.48	1.171 ± 0.014	1.67 ± 0.69	1.169 ± 0.012	1.74 ± 0.69
**3**	1.181 ± 0.009	0.91 ± 0.55	1.176 ± 0.007	1.07 ± 0.48	1.17 ± 0.009	1.50 ± 0.38
**6**	1.186 ± 0.015	0.22 ± 0.68	1.177 ± 0.009	0.87 ± 0.46	1.166 ± 0.008	1.66 ± 0.29
**8**	1.187 ± 0.007	0.02 ± 0.55	1.176 ± 0.009	0.79 ± 0.52	1.161 ± 0.011	2.08 ± 0.45
**9**	1.186 ± 0.009	0.04 ± 0.55	1.176 ± 0.011	0.82 ± 0.42	1.158 ± 0.016	2.29 ± 0.88
**10**	1.184 ± 0.011	0.16 ± 0.58	1.174 ± 0.013	0.96 ± 0.65	1.155 ± 0.014	2.52 ± 0.71
**Dry phase**						
**10.25**	1.166 ± 0.009	2.01 ± 0.44	1.155 ± 0.007	2.85 ± 0.42	1.135 ± 0.013	4.51 ± 0.52
**13**	1.142 ± 0.011	4.38 ± 0.48	1.132 ± 0.013	5.20 ± 0.59	1.11 ± 0.018	7.07 ± 0.68
**15**	1.135 ± 0.009	5.13 ± 0.55	1.127 ± 0.012	5.81 ± 0.58	1.105 ± 0.009	7.60 ± 0.61
**17**	1.132 ± 0.008	5.48 ± 0.58	1.124 ± 0.011	6.08 ± 0.49	1.101 ± 0.010	8.13 ± 0.56
**20**	1.129 ± 0.007	5.84 ± 0.55	1.124 ± 0.006	6.23 ± 0.52	1.101 ± 0.009	8.22 ± 0.45
**24**	1.127 ± 0.009	6.03 ± 0.55	1.122 ± 0.013	6.51 ± 0.65	1.104 ± 0.009	8.00 ± 0.48
**28**	1.128 ± 0.011	5.97 ± 0.58	1.125 ± 0.009	6.30 ± 0.32	1.109 ± 0.014	7.61 ± 0.49

## Data Availability

The original contributions presented in the study are included in the article, further inquiries can be directed to the corresponding author.
